# *GSTD1* Mediates the Tolerance to Abamectin and Beta-Cypermethrin in the Fall Armyworm *Spodoptera frugiperda*

**DOI:** 10.3390/insects16030299

**Published:** 2025-03-12

**Authors:** Qian Ding, Yangyang Liu, Baoxiang Dai, Yujie Han, Yan Zhang, Zhongyuan Deng, Lixiang Wang, Xianchun Li

**Affiliations:** 1 Zhengzhou Research Base, State Key Laboratory of Cotton Biology, School of Agricultural Sciences, Zhengzhou University, Zhengzhou 450001, China; 2 Key Laboratory of Sustainable Management of Forest Ecosystem, Northeast Forestry University, Harbin 150040, China; 3 Department of Entomology, BIO5 Institute, University of Arizona, Tucson, AZ 85721, USA

**Keywords:** *Spodoptera frugiperda*, *SfGSTD1*, abamectin, beta-cypermethrin, insecticide tolerance, detoxification

## Abstract

Glutathione S-transferase (GST) is a class of detoxifying enzymes in the second stage of insect metabolism and plays a key role in insecticide resistance. In this study, based on the transcriptome sequences of *S. frugiperda*, the full-length cDNA of *SfGSTD1* was cloned and characterized. The temporal and spatial expression pattern showed that *SfGSTD1* was highly expressed in Malpighian tubules, which are key excretion organs. Knocking down *SfGSTD1* reduced *S. frugiperd* tolerance to abamectin and beta-cypermethrin. The overexpression of *SfGSTD1* enhanced the viability of *Sf9* cell under abamectin and beta-cypermethrin treatment. Furthermore, *SfGSTD1* was overexpressed in *Drosophila melanogaster* using the *GAL4*/*UAS* binary expression system, and this overexpression strain was also less susceptible to abamectin and beta-cypermethrin. The enzyme activity of recombinant SfGSTD1 could also be significantly inhibited by abamectin and beta-cypermethrin. Taken together, our findings indicate that *SfGSTD1* might be involved in the tolerance of abamectin and beta-cypermethrin in *S. frugiperda*. And these results provide theoretical foundations for understanding the resistance mechanism of *S. frugiperda* to abamectin and beta-cypermethrin.

## Simple Summary

*Spodoptera frugiperda* is a notorious pest native to subtropical and tropical regions of the Americas, and it can cause serious harm to many economic crops. Although the use of chemical insecticides is the most effective and quickest way to control *S. frugiperda*, it inevitably leads to the development of insect resistance. *GSTD1* has been reported to be involved in the detoxification of insects towards insecticides. In this study, we cloned and identified the *SfGSTD1* gene in *S. frugiperda*. To verify whether *SfGSTD1* mediates the tolerance of *S. frugiperda* to insecticides, we firstly used RNAi technology to knock down the *SfGSTD1* gene, and then analyzed the changes in insecticide tolerance of *S. frugiperda* to two insecticides (abamectin and beta-cypermethrin). Subsequently, we further validated the gene function of *SfGSTD1* by overexpressing it in *Sf9* cells and *Drosophila melanogaster*. Finally, the inhibition assay was carried out to explore the potential interactions between SfGSTD1 and insecticides (abamectin and beta-cypermethrin). Our results showed that *SfGSTD1* is closely related to the detoxification of abamectin and beta-cypermethrin, and suggested that the abamectin and beta-cypermethrin resistance of *S. frugiperda* may be related to *SfGSTD1* overexpression.

## 1. Introduction

The fall armyworm, *Spodoptera frugiperda* (Lepidoptera: Noctuidae), is one of the most devastating pests worldwide and can cause severe crop and economic loss. It is a highly polyphagous pest, and its hosts range over 350 species across 76 plant families including maize, sorghum, soybean, cotton, rice and wheat [1,2,3,4]. The chemical control of *S. frugiperda* has a long history, and a variety of insecticides have been used for its prevention and control, with field populations in many countries developing varying degrees of resistance to traditional pesticides. The first report on the chemical resistance of *S. frugiperda* was in 1979; a field population in Georgia (USA) exerted a certain level of resistance to carbaryl [5]. Its resistance to trichlorfon, pyrethroids, carbamates and organophosphorus insecticides was reported soon thereafter [6,7]. Since then, *S. frugiperda* has evolved resistance to a minimum of 47 active ingredients in different chemical classes from the arthropod pesticide resistance database (APRD, 2025, https://www.pesticideresistance.org/). Due to past selection by insecticides, a single *S. frugiperda* population could evolve multiple resistance; for example, a population in Puerto Rico was found to have resistance against 10 commonly used pesticides [8]. The study of resistance mechanisms of *S. frugiperda* is the basis of field-integrated pest management. Metabolic resistance mediated by detoxification enzymes is one of the most important mechanisms of multidrug resistance generation.

Glutathione-S-transferase (GST) is a multifunctional enzyme family present in most living organisms, and plays a very important physiological roles including immune response, antioxidation, odorant degradation, xenobiotic resistance and ecdysteroid biosynthesis [9,10,11,12]. GST is one of the most important families of detoxification enzymes. The research on insect GSTs mainly focuses on the detoxification of electrophilic substances such as insecticides, toxic secondary metabolites and endogenous toxic substances. GST can participate in the formation and development of insecticide resistance through glutathione conjugation, noncatalytic binding, antioxidant stress response and directly catalyzing the metabolism [13]. The overexpression of GST mediates enhanced detoxification, which has been implicated in the formation and development of insecticide resistance. For example, in *Helicoverpa armigera*, the overexpression of *HaT_119* and *HaGST_121* were significantly linked to lambda-cyhalothrin resistance, whereas the upregulation of *HaGSTS1* and *HaGSTD1* participated in malathion resistance [14,15]. In *Nilaparvata lugens*, the overexpression of *NlGSTS1* and *NlGSTS2* was associated with organophosphates and synthetic pyrethroids resistance [16]. In addition, qualitative changes in GST were also reported to have evolved in insecticide resistance, such as a single mutation V128A in the *BdGSTE8* which confers malathion resistance in *Bactrocera dorsalis* [17].

Insect GSTs are mainly divided into seven subfamilies, including delta, epsilon, omega, sigma, theta, zeta and microsomal, among which the delta and epsilon are the most abundant and are unique to insects. Extensive studies have documented the detoxification activity of GSTs in various insect species, with particular emphasis on the detoxification ability of the Delta family to insecticides. In *Bradysia odoriphaga*, *BoGSTD2* exhibits peroxidase activity, preventing oxidative stress caused by exposure to malathion and thiamethoxam [18]. In *Cydia pomonella*, lambda-cyhalothrin could be significantly metabolized by recombinant *CpGSTD1*, indicating that *CpGSTD1* may be involved in the formation of cyhalothrin resistance [19]. *TuGSTD01* in *Tetranychus urticae* was significantly inhibited by abamectin but could not detoxify abamectin, which indicates that it may undergo noncatalytic binding with abamectin to reduce its toxicity [20]. *TcGSTD2* from *Tribolium castaneum* has been reported, which can detoxify insecticides by combating oxidative stress [21]. In terms of the GST having various mechanisms of interaction with insecticides, exploring their important roles in detoxifying insecticides helps in understanding the mechanisms of insecticide resistance and cross resistance.

To elucidate whether GSTs are involved in abamectin and beta-cypermethrin detoxification in *S. frugiperda*, a delta class GST gene *SfGSTD1* was cloned for functional verification in this study. The expression pattern of *SfGSTD1* in different tissues and developmental stages was analyzed via quantitative real-time PCR. The susceptibility of *S. frugiperda* to abamectin and beta-cypermethrin after knocking down and *SfGSTD1* was further analyzed to investigate the role of *SfGSTD1* in insecticide detoxification. Moreover, the function of *SfGSTD1* was also determined through a *GAL4/UAS* binary expression system in *D. melanogaster* and the *piggyBac* overexpression system in the *Sf9* cell line. We successfully purified and validated the recombinant proteins through a prokaryotic expression system and then characterized their biochemical properties, including kinetic parameters and insecticide inhibition, using spectrophotometric microplate assays. These results demonstrate that *SfGSTD1* plays a crucial role in the detoxification of *S. frugiperda* to abamectin and beta-cypermethrin.

## 2. Materials and Methods

### 2.1. Insects and Insecticides

The *S. frugiperda* strain used in this research was kindly supplied by Dr. Shao-Hua Gu from the School of Plant Protection, China Agricultural University, and was originally collected from a maize field (Kunming, China) in June 2019. The adult was fed with 10% sucrose water and laid eggs on cotton gauze. The newly hatched larvae were kept on a semisynthetic diet at 27 ± 1 °C and relative humidity at 75 ± 5% with a 16 h:8 h light/dark photoperiod. The pupae were disinfected with 0.25% sodium hypochlorite solution and placed under the same environmental conditions for emergence.

Abamectin (96.8%) was procured from Hebei Weiyuan Biochemical Co., Ltd. (Shijiazhuang, China). Beta-cypermethrin (95%) was procured from Jiangsu Gongcheng Biotechnology Co., Ltd. (Nanjing, China). Abamectin and beta-cypermethrin technical-grade insecticides were dissolved in dimethyl sulfoxide and acetone, respectively, to form the solution of 50,000 mg/L.

### 2.2. RNA Isolation and cDNA Synthesis

Total RNA was extracted by employing the RNAiso Plus reagent (Takara, Dalian, China) according to the instructions. The integrity and concentration of the obtained total RNA were determined through the use of agarose gel electrophoresis, along with a Nanodrop2000 spectrophotometer (Thermo Fisher Scientific, Wilmington, DE, USA). First-strand cDNA was synthesized by using a HiScript II 1st Strand cDNA Synthesis Kit (+gDNA wiper) (Vazyme, Nanjing, China). The cDNA and RNA samples were stored at −40 °C and −80 °C, respectively.

### 2.3. Sequence Analysis and Phylogenetic Tree Construction

**Figure 1:**
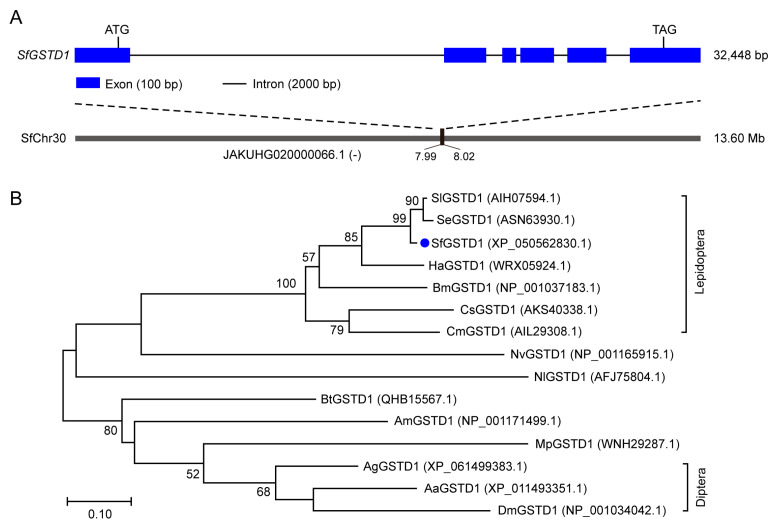
Sequence analysis of *SfGSTD1* gene. (**A**) Gene structure of *SfGSTD1* gene. The exons are depicted as blue boxes, while the introns are represented by black lines connecting the boxes. The intron-to-exon ratio was determined to be 20:1. The predicted start codon (ATG), stop codon (TAG) and the chromosomal location of gene locus (with “-” indicating the reverse orientation with chromosome) are annotated at their respective positions. (**B**) Phylogenetic tree of the *SfGSTD1* gene. The phylogenetic tree is constructed using maximum likelihood method. The number above the branch indicates support for the phylogenies and only displays values greater than 50. Accession numbers of the used sequences are listed after the corresponding genes. Aa, *Aedes aegypti*; Ag, *Anopheles gambiae*; Am, *Apis mellifera*; Bm, *Bombyx mori*; Bt, *Bemisia tabaci*; Cm, *Cnaphalocrocis medinalis*; Cs, *Chilo suppressalis*; Dm, *Drosophila melanogaster*; Ha, *Helicoverpa armigera*; Mp, *Myzus persicae*; Nl, *Nilaparvata lugens*; Nv, *Nasonia vitripennis*; Se, *Spodoptera exigua*; Sf, *Spodoptera frugiperda*; Sl, *Spodoptera litura*.

Based on the *S. frugiperda* transcriptome, the sequence of *SfGSTD1* (GeneBank: XP_050562830.1) was amplified using 2 × Phanta Flash Master Mix (Vazyme, Nanjing, China) and specific primers (Table S1). The PCR product was then linked to the pMD™19-T Vector (TaKaRa, Beijing, China) for sequencing validation. The open reading frame (ORF), amino acid sequence, and sequence alignment were performed on the obtained *SfGSTD1* clones using DNAMAN version 8.0 and ESPript 3.0. The protein isoelectric point (*pI*) and molecular mass were predicted using the Expasy Proteomics web tool https://web.expasy.org/protparam/, accessed on 5 March 2024). The phylogenetic tree was generated using the maximum likelihood (ML) method implemented in MEGA 7.0 software [22]. Jones–Taylor–Thornton matrix-based mode with 2000 bootstrap replicates was implemented for phylogenetic tree analysis. Bootstrap values higher than 50% and the GenBank accession numbers of sequences used in the phylogenetic analyses are given in Figure 1.

### 2.4. Real-Time Quantitative PCR Analyses (RT-qPCR)

The gene expression pattern at different development stages and various tissues was investigated. In different development stages, eggs, 1st- to 6th-instar larvae, pupae and adults (male and female) were sampled separately. Various tissues, including the head (HD), midgut (MG), Malpighian tubules (MT), fat body (FB), hemolymph (HE) and integument (IN), were dissected from 6th larvae (*n* = 60). These dissected samples were temporarily kept in cold RNAiso Plus reagent (Takara, Dalian, China) and then subjected to RNA extraction according to the instructions. The cDNA was synthesized using the HiScript II Q Select RT SuperMix for qPCR kit (Vazyme, Nanjing, China) in a 20 μL reaction volume with 1 μg of RNA. Specific RT-qPCR primers (Table S1) were designed using Primer3 software (http://bioinfo.ut.ee/primer3/, accessed on 10 June 2024). The qPCR analyses were carried out using the QuantStudio 5 system (Applied Biosystems, Foster City, CA, USA) with UltraSYBR Mixture (with ROX) Kit (CWBIO, Beijing, China). Each qPCR reaction contained 4 μL of cDNA sample, 1 μL of forward primer (10 μM), 1 μL of reverse primer (10 μM), 4 μL of RNase-free water and 10 μL of UltraSYBR mixture. The program parameters were set as follows: initial denaturation at 95 °C for 10 min, followed by 40 cycles of denaturation at 95 °C for 15 s and annealing/extension at 60 °C for 40 s. A final melt curve stage was implemented to assess the specificity of primer amplification. Each primer pair’s amplification efficiency was counted by a serial dilution of cDNA (0.01 ng to 100 ng). Each sample had at least three biological replicates. The ribosomal protein L32 (*RPL32*) and glyceraldehyde-3-phosphate dehydrogenase (*GADPH*) were selected as housekeeping genes for the normalization of target gene mRNA levels. Relative gene expression was calculated using the 2^−ΔΔCT^ method [23].

### 2.5. Preparation of dsRNA and RNA Interference

An approximately 442 bp fragment of *SfGSTD1* was amplified (Table S1) and ligated into the pET-2P vector constructed from pET-30a (+) [24]. A 428 bp fragment of *EGFP* was also amplified and ligated into pET-2P as a negative control. The recombinant plasmids were transferred into *Escherichia coli* HT115 (DE3) competent cells to synthesis dsRNA. The positive transformants were cultured in 500 mL Luria broth medium containing 50 μg/mL kanamycin at 37 °C with shaking at 220 rpm. The bacteria was induced with a 0.5 mM final concentration of isopropyl-β-D-thiogalactopyranoside (IPTG) when OD_600_ reached 1.0, followed by shaking at 220 rpm for an additional 4 h at 37 °C. The bacterial cells were harvested by centrifugation at 8000 rpm for 10 min, and the pellet was weighed and resuspended in 0.05 M phosphate-buffered saline (pH = 7.4). The expressed dsRNA was extracted from *E. coli* and validated by electrophoresis on a 1% agarose gel.

A diet incorporation bioassay was implemented to knock down the expression level of *SfGSTD1*. Firstly, the *E. coli* expressing dsRNA were mixed for an artificial diet at a concentration of 3 mg/g. The dsRNA diet was added to 24-well plates and fed to 3^rd^-instar larvae of *S. frugiperda*. To verify the RNAi efficiency, the *SfGSTD1* transcription levels in the whole larval body were analyzed daily by qPCR. Subsequently, 200 mg/L abamectin or 25 mg/L beta-cypermethrin were added to an artificial diet that contained dsRNA. The mortality of *S. frugiperda* was determined at 72 h after insecticide treatment. The bioassays were performed with three biological replicates, and 20 larvae were used in each bioassay.

### 2.6. Construction of Sf9 Cell Lines Stably Expressing SfGSTD1

*S. frugiperda* ovarian cells (*Sf9* cells) were cultured in SF-900TM II medium (Gibco, Gaithersburg, MD, USA) supplemented with 100 units/mL penicillin, 100 mg/mL streptomycin and 10% heat-inactivated fetal bovine serum and maintained at 27 °C. In this study, the *piggyBac* vector system was adopted to construct stable expression *Sf9* cell lines that overexpressed *SfGSTD1* according to the method we previously used [25]. The *piggyBac:SfPub/P2009-target:hr5/OpIE1-EGFP-PuroR* vector was constructed using piggyBac vector (Miaoling Biology, Wuhan, China), as described in Chen’s research [26]. Then, 1 μg of constructed *piggyBac* vector and 1 μg transposase plasmid were mixed in equal proportions and transfected into cells using the FuGENE^®^ HD reagent (Promega, Madison, WI, USA) in a 6-well plate. Next, 10 µg/mL puromycin was used to selected recombination *Sf9* cells, and the expression level of the *SfGSTD1* was validated using RT-qPCR after three weeks selection. The *EGFP* overexpressed cell line was used as the control.

### 2.7. Cell Viability Assay

The Cell Counting Kit-8 (CCK-8, Yeasen Biotechnology, Shanghai, China) was used to determine the viability of cells according to the instructions. Firstly, 5 × 10^4^  *Sf9* cells were seeded into each well of the 96-well plate. The final concentration of 20 mg/L abamectin and 60 mg/L beta-cypermethrin diluted with culture medium (10 μL) was added to each well after 2 h. The *Sf9* cells treated with insecticide were then cultivated for 2 d. Finally, CCK-8 solution (10 μL/well) was added to the culture medium and incubated for 2 h again.

The absorbance at 450 nm was determined using a Spark multimode microplate reader (Tecan, Switzerland). Each treatment had at least three biological replicates. The calculation formula for relative cell activity is as follows:

$$Cell\;viability\;(\%)\;=\;\frac{OD\;value\;of\;treatment\;group-OD\;value\;of\;blank\;group}{OD\;value\;of\;control\;group-OD\;value\;of\;blank\;group}\times 100$$

### 2.8. Construction of Transgenic D. melanogaster and Bioassay

The ORF of *SfGSTD1* was ligated to *pJFRC2-10XUAS-IVS-mCD8::GFP* plasmid (Addgene: 26214) for the *UAS*-*SfGSTD1* construction of transgenic flies. The eggs containing PhiC31 transposase and the *attP* sequence were subsequently collected for injection by the Qidong Fangjing Biotechnology Co., Ltd. (Nantong, Jiangsu, China). After microinjection, F0 male flies were first hybridized with the wild-type strain (*W^1118^*), and F1 male flies were selected again to hybridize with the balancer (*W*^−^;;*MKRS/TM6B*) to remove PhiC31 transposase. Homozygous strains of *UAS*-*SfGSTD1* were obtained by self-crossing. The *Actin-GAL4* strain (Bloomington Drosophila Stock Center: 30558) was employed to drive the expression of *SfGSTD1* throughout the whole body of *D. melanogaster*. The expression level of the *SfGSTD1* in transgenic *D. melanogaster* was verified by RT-PCR.

A bioassay was used to determine the susceptibility of female adults that had emerged for 3-6 days to insecticides. Here, 100 mg/L abamectin and 10 mg/L beta-cypermethrin were prepared with 5% sucrose and 1% agar, and air-dried overnight. Fifteen female flies were transferred to each vial containing the aforementioned toxic diet, and the mortality rate was counted after 72 h. At least four biological replicates were carried out for each genotype of *D. melanogaster*.

### 2.9. Expression and Purification of Recombinant Proteins

The verified *SfGSTD1* ORF was amplified using primers (Table S1) containing homologous sequences and subsequently cloned into the *pCold II* expression vector (Takara, Dalian, China). The recombinant plasmid was then transformed into BL21 (DE3) competent cells. Positive transformants were selected and cultured in 500 mL of LB medium supplemented with 100 μg/mL ampicillin at 37 °C with constant shaking at 220 rpm. The bacterial culture was maintained at 15 °C for 30 min to allow temperature adaptation and then induced with isopropyl β-D-thiogalactopyranoside (IPTG) to a final concentration of 0.75 mM, followed by incubation at 15 °C with shaking at 160 rpm for 12 h. The bacterial cells were harvested by centrifugation at 10,000 rpm for 10 min at 4 °C, and the pellet was resuspended in 10 mL of ice-cold lysis buffer supplemented with imidazole (10 mM) and lysozyme (1 mg/mL). After sonication and centrifugation (12,000 rpm, 30 min, 4 °C), the supernatant was purified using Ni-NTA columns (Sangon, Shanghai, China). The recombinant protein was then purified according to the protocol and detected using 8% SDS-PAGE. The purified target protein was dialyzed overnight in Tris-HCl buffer (25 mM, pH 7.4). The concentration of purified SfGSTD1 protein was quantified by the BCA assay [27].

### 2.10. Enzyme Kinetic Properties and Enzyme Inhibition Assays

The kinetic parameters of recombinant protein were evaluated using 1-chloro-2-dinitrobenzene (CDNB) and GSH as substrates, and the absorbance was monitored using a microplate reader SpectraMax PLUS384 (Molecular Devices, San Jose, CA, USA). The 200 μL reaction solution contained 1 μg of recombinant protein, CDNB (0.125 to 4 mM) and 4 mM GSH. The absorbance of the reaction mixture was detected at 340 nm. The heated protein was used in place of the recombinant protein as the control, and each assay was performed in triplicate. The *K_m_* and *V_max_* values were determined using GraphPad Prism 9 (Graphpad, San Diego, CA, USA).

The half maximal inhibitory concentrations (IC_50_) were assayed by pre-incubating 1 μg recombinant protein with a range of concentration of 0.0018 to 0.114 mM for abamectin and 0.0038 to 0.240 mM for beta-cypermethrin that included seven concentrations. The residual activity of the protein was determined according to the method mentioned above.

### 2.11. Statistics

Statistical analysis was performed using a two-tailed unpaired Student’s *t*-test and one-way ANOVA with Tukey’s multiple range test for post hoc comparisons. Significance was set at * *p* < 0.05, ** *p* < 0.01 and *** *p* < 0.001. GraphPad Prism 9.0 software (San Diego, CA, USA) was employed to draw the histograms containing the mean and standard error of the mean (SEM).

## 3. Results

### 3.1. Molecular Characterization of SfGSTD1

The cDNA sequence of *SfGSTD1* (GeneBank: XP_050562830.1) was characterized from *S. frugiperda*. The complete ORF of *SfGSTD1* with 735 bp was amplified by PCR, which encodes a 244 amino acid residue protein. The molecular weight of *SfGSTD1* was predicted as 27.89 kDa and the theoretical pI point as 5.48. No signal peptide, transmembrane domain and N-glycosylation site were predicted in *SfGSTD1*. The GSH-binding site (G-site) and the hydrophobic substrate-binding site (H-site) of *SfGSTD1* were predicted, which included 6 and 10 amino acids separately (Figure S1). According to the secondary structure of protein prediction results, *SfGSTD1* has nine α-helixes and four β-sheets. The alignment between *SfGSTD1* and the genome sequence of *S. frugiperda* showed that *SfGSTD1* has six exons and four introns, located in chromosome 30 (JAKUHG020000066.1) with the reverse orientations (Figure 1A). To analyze the evolutionary relationship of *SfGSTD1*, a phylogenetic tree was constructed with orthologs from 15 insect species. The phylogenetic tree comparison showed that *SfGSTD1* clustered with *GSTD1* from other Lepidoptera insects, and has a slightly distant homologous relationship with Diptera insects (Figure 1B).

### 3.2. Temporal and Spatial Expression of SfGSTD1

**Figure 2:**
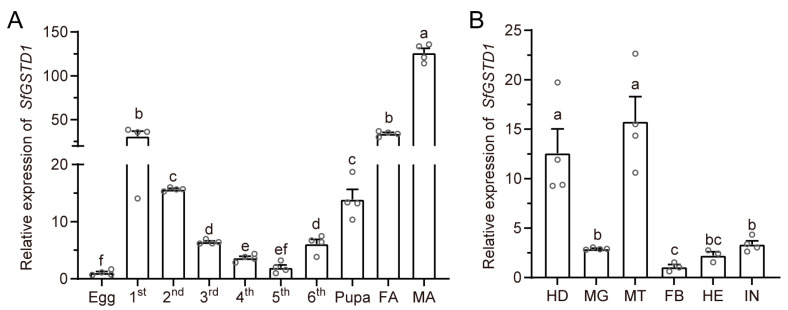
The spatiotemporal expression of *SfGSTD1*. (**A**) Expression levels of *SfGSTD1* in different developmental stages, encompassing the egg stage, 1st- to 6th-instar larvae stages, pupal stage, female adult (FA) and male adult (MA). (**B**) The expression levels of *SfGSTD1* in various tissues, including the head (HD), midgut (MG), fat body (FB), Malpighian tubules (MT), integument (IN) and hemolymph (HE). Each circle represents a specific value for each repetition and all data are presented as the mean ± SEM (*n* > 3). Significant differences (*p* < 0.05) among groups, determined by one-way ANOVA followed by Tukey’s multiple comparison test, are indicated by different lowercase letters above the columns.

*SfGSTD1* was expressed in different developmental stages and various tissues. In different developmental stages of *S. frugiperda*, the expression level of *SfGSTD1* was the lowest during the egg stage, gradually decreasing from 1st to 5^th^ instar, and then reaching the highest in the adult stage (Figure 2A). Tissues expression results showed that *SfGSTD1* was predominately expressed in Malpighian tubules and head, followed by midgut and integument, while the fat body was the tissue with the lowest expression level (Figure 2B).

### 3.3. Silencing of SfGSTD1 Increased Larval Susceptibility of S. frugiperda to Insecticides

**Figure 3:**
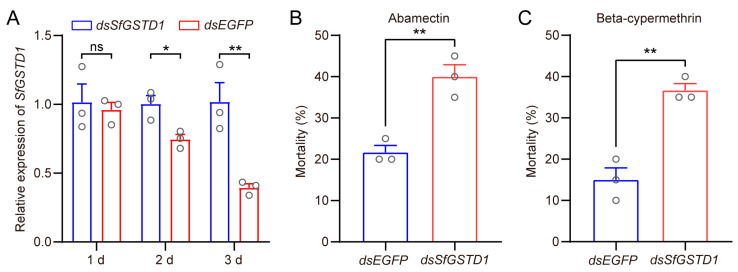
Functional analysis of *SfGSTD1* in *S. frugiperda* by RNAi followed by insecticide bioassay. (**A**) Relative expression level of *SfGSTD1* was assessed at 1–3 days after feeding on artificial diet supplemented with dsRNA (*dsSfGSTD1* and *dsEGFP*). (**B**) The mortality rate of larvae with *SfGSTD1* gene knocked down to 200 mg/L abamectin. (**C**) The mortality rate of larvae with *SfGSTD1* gene knocked down to 25 mg/L beta-cypermethrin. The mortality was recorded after feeding with a toxic diet containing abamectin or beta-cypermethrin for 3 days. At least three replicates were carried out, each circle represents a specific value for each repetition and the bar represents means ± SEM (Student’s *t*-test, ns: not significant, * *p* < 0.05 and ** *p* < 0.01).

To evaluate whether *SfGSTD1* was involved in the tolerance of *S. frugiperda* to insecticides, we performed RNAi technology to knock down the expression level of *SfGSTD1*. These results showed that compared with larvae feeding with the artificial diet containing *dsEGFP*, the expression level of *SfGSTD1* did not significantly change after feeding with an artificial diet containing *dsSfGSTD1* for 1 d, but significantly decreased after 2–3 d (Figure 3A). Subsequently, a diet incorporation bioassay was implemented to evaluate the tolerance of *S. frugiperda* to two insecticides after *SfGSTD1* was knocked down. The mortality of *S. frugiperda* larvae fed on *dsSfGSTD1* was significantly increased to 40.0% after being exposed to abamectin compared to larvae fed on *dsEGFP* (21.7%) (Figure 3B). Similarly, the silencing of *SfGSTD1* could increase the mortality of *S. frugiperda* exposed to 25 mg/L beta-cypermethrin (Figure 3C).

### 3.4. Overexpression of SfGSTD1 Decreased Toxicity of Insecticides to Sf9 Cells

**Figure 4:**
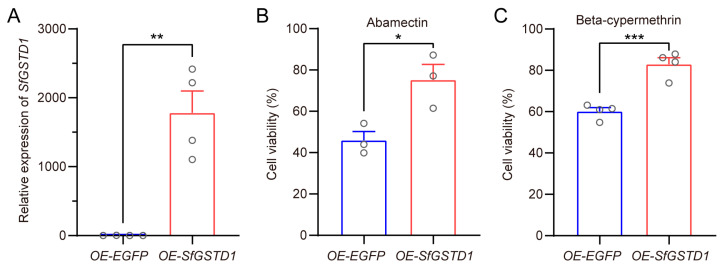
Toxicity analysis of abamectin and beta-cypermethrin on *Sf9* cells overexpressing-*SfGSTD1* (*OE*-*SfGSTD1*). (**A**) The expression level was detected by qPCR in *Sf9* cells overexpressing *SfGSTD1* and *EGFP*. (**B**) The relative viability of *Sf9* cells (*OE*-*SfGSTD1* and *OE-EGFP*) after exposure to 20 mg/L abamectin. (**C**) The relative viability of *Sf9* cells (*OE*-*SfGSTD1* and *OE-EGFP*) after exposure to 60 mg/L beta-cypermethrin. At least three replicates were carried out, each circle represents a specific value for each repetition and the bar represents means ± SEM (Student’s *t*-test, * *p* < 0.05, ** *p* < 0.01 and *** *p* < 0.001).

We utilized the *piggyBac* transposon to stably express *SfGSTD1* in *Sf9* cells to investigate its function. The qPCR results showed that compared with the negative control group that only overexpressed *EGFP*, the expression level of the *SfGSTD1* gene was significantly upregulated in stable overexpressing *SfGSTD1* cell lines (Figure 4A). The relative viability of *SfGSTD1* and *EGFP* overexpressing cells was subsequently measured after treatment with abamectin and beta-cypermethrin. Compared to *EGFP* overexpressing *Sf9* cells, the overexpression of *SfGSTD1* significantly enhanced the cell viability of *Sf9* cells exposed to abamectin and beta-cypermethrin, by 29.2% (from 46.0% to 75.2%) and 22.9% (from 60.1% to 83.0%) (Figure 4B,C).

### 3.5. Expression of SfGSTD1 in D. melanogaster Leads to Decreased Susceptibility to Insecticides

**Figure 5:**
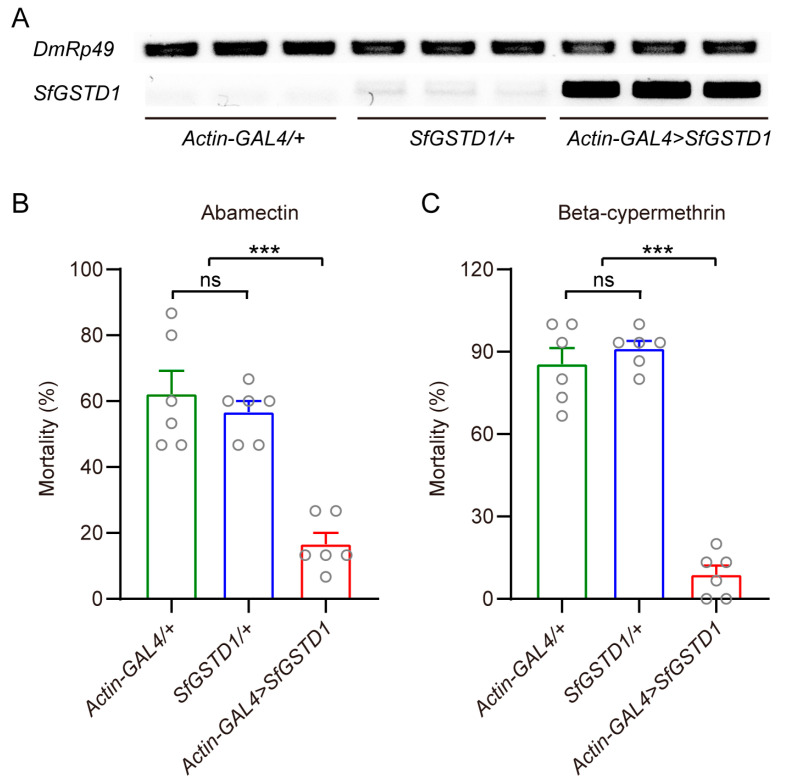
Transgenic expression of *SfGSTD1* in *D. melanogaster* and the effects on abamectin and beta-cypermethrin tolerance. (**A**) The expression of *SfGSTD1* was confirmed by RT-PCR in control strain (*Actin-GAL4*/+ and *SfGSTD1*/+) and transgenic flies overexpressing *SfGSTD1* (*Actin-GAL4* > *SfGSTD1*). (**B**) The mortality rate of *D. melanogaster* overexpressing *SfGSTD1* to 100 mg/L abamectin. (**C**) The mortality rate of *D. melanogaster* overexpressing *SfGSTD1* to 10 mg/L beta-cypermethrin. Each circle represents a specific value for each repetition and data are expressed as mean ± SEM (Student’s *t*-test, ns: not significant, *** *p* < 0.001).

To further verify the function of the *SfGSTD1* gene in the insecticide’s detoxification, we constructed a *UAS*-*SfGSTD1* transgenic fly strain. The *Actin*-*GAL4* strain was employed to drive the overexpression of *SfGSTD1* in *D. melanogaster*, and this result was validated through semi-quantitative analysis (Figure 5A). The mortalities of female adults treated with abamectin and beta-cypermethrin were then recorded. Bioassay results showed that the mortality of transgenic flies overexpressing *SfGSTD1* was significantly reduced by 40.0% after exposure to abamectin compared to the control flies (*SfGSTD1/+*) (Figure 5B). Moreover, the overexpression of *SfGSTD1* could significantly decrease the susceptibility of *D. melanogaster* exposed to 10 mg/L beta-cypermethrin (Figure 5C).

### 3.6. Enzymatic Properties and Inhibitions of Insecticides on Recombinant SfGSTD1

**Figure 6:**
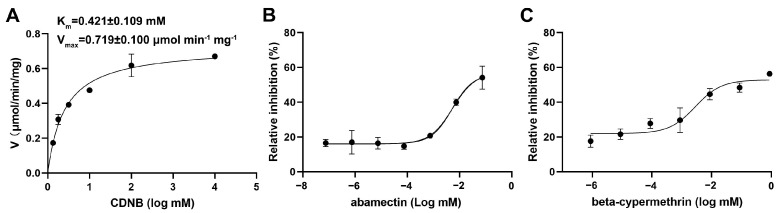
Enzyme properties of recombinant SfGSTD1 and inhibitions of abamectin and beta-cypermethrin on SfGSTD1. (**A**) Enzymatic kinetic parameters of recombinant SfGSTD1. (**B**) Dose−response curves for the inhibitions of abamectin on the CDNB conjugating activity of recombinant SfGSTD1. (**C**) Dose−response curves for the inhibitions of beta-cypermethrin on the CDNB conjugating activity of recombinant SfGSTD1.

To assess the catalytic activity of SfGSTD1, the recombinant protein with an N-terminal His-tag was expressed in *E. coli* BL21 (DE3) and purified through the Ni-NTA spin column. SDS-PAGE analysis revealed a single band at approximately 27 kDa (Figure S2), corresponding to the predicted size of SfGSTD1 (27.89 kDa) plus the His-tag (0.97 kDa). Kinetic analysis using CDNB and GSH as substrates revealed that recombinant SfGSTD1 exhibited a *K*_m_ value of 0.421 ± 0.109 mM and a Vmax value of 0.719 ± 0.100 μmol/min/mg to CDNB (Figure 6A).

Subsequently, the inhibition assay was determined to explore the potential interactions between SfGSTD1 and two insecticides. The result revealed that the enzyme activity of SfGSTD1 was significantly inhibited by abamectin and beta-cypermethrin (Figure 6B,C). Low concentrations of abamectin (0.002–0.057 mM) showed relatively weak inhibitions to SfGSTD1, while the inhibition was remarkably increased to 54.17% when 0.458 mM of abamectin was used. The inhibitions of beta-cypermethrin on SfGSTD1 activity were raised with the increase in insecticide concentrations, and relative inhibition reached 56.39% when 0.960 mM beta-cypermethrin was used. The abamectin and beta-cypermethrin showed the inhibition on SfGSTD1 with the half-inhibitory concentration (IC_50_) of 0.208 mM and 0.171 mM, separately.

## 4. Discussion

GST, as an important detoxifying enzyme, is widely involved in the detoxification of insects towards insecticides and plant secondary metabolites. This metabolic mechanism ensures that insects can adapt to exposure to external insecticides [28]. In this study, a GST gene was cloned from *S. frugiperda*, which belongs to the delta subfamily. Similar to the GST family genes of other insects [29], *SfGSTD1* has two conserved GST-signature motifs; one is the GSH-binding site (G-site) and the other is the hydrophobic substrate-binding site (H-site) (Figure S1). These play a critical role in insecticide metabolism [30]. In addition, *SfGSTD1* has nine α-helixes and four β-sheets, according to the predicted protein secondary structure. The homologous gene of *SfGSTD1* has also been found in multiple other insects, including Lepidoptera, Hemiptera, Diptera and Hymenoptera. Evolutionary tree analysis showed that *SfGSTD1* was closely clustered with homologous genes in the Lepidoptera of *S. exigua* and *S. litura* (Figure 1B). BLASTP analysis showed that SfGSTD1 shares 96.5%, 95.8%, 73.3%, 51.3%, 48.9%, 48.2% and 44.1% sequence identity with the SfGSTD1 of *S. litura*, *S. exigua*, *C. medinalis*, *A. gambiae*, *B. tabaci*, *D. melanogaster* and *N. lugens*, respectively. Thus, the *SfGSTD1* was characterized and bioinformatic analysis performed from different levels and perspectives.

The temporal and spatial expression pattern showed that *SfGSTD1* was expressed in different developmental stages and various tissues, suggesting that *SfGSTD1* may be involved in many physiological functions. During the developmental stages from 1^st^-instar larvae to adult, the expression level of *SfGSTD1* showed a trend of first decreasing and then increasing, and there were significant differences between male and female adults (Figure 2A). This result indicates that *SfGSTD1* may also have other physiological functions, such as regulating the reproductive behavior of *S. frugiperda*, but more work is needed to uncover these physiological roles. The Malpighian tubules are well known as the essential excretory tissues in most insects [31], and we found that *SfGSTD1* was predominantly expressed in these tissues (Figure 2B). Consistent with our results, *BdGSTD1* and *BdGSTD10* also exhibited high expression levels in the Malpighian tubules of *B. dorsalis* [32]. In addition, *SfGSTE9*-mediated beta-cypermethrin tolerance in *S. frugiperda* was observed to be the most abundant in the head, early larval stages and pupae [33]. Based on these research results, it could be inferred that some insects’ GST associated with insecticide resistance are not only most abundant in detoxifying tissues and larvae stages, but also highly expressed in other developmental stages such as pupae and adults.

GST genes in delta and epsilon subfamilies have been widely reported to mediate insecticide resistance in various insects such as *B. dorsalis*, *S. litura* and *S. exigua* [32,34,35,36,37]. Among numerous members of the delta class, *GSTD1* has been shown to be involved in the resistance of insects to various insecticides. In *Periplaneta americana*, *PaGSTD1* has been verified as an important detoxifying enzyme with high metabolic efficiency against chlorpyrifos-methyl [38]. In *C. pomonella*, *CpGSTD1* was not only upregulated after treatment with lambda-cyhalothrin, but also significantly metabolized lambda-cyhalothrin [19]. Metabolism assays showed that malathion could be significantly depleted by BdGSTD1 of *B. dorsalis* [32]. Moreover, *SlGSTD1* is associated with the resistance of *S. litura* to cyhalothrin and fenvalerate by antioxidant capacity and detoxication [37]. In *C. suppressalis*, *CsGSTD1* might confer tolerance to abamectin through the noncatalytic passive binding and sequestration [39]. Here, we confirmed the *SfGSTD1*-mediated tolerance of *S. frugiperda* to two insecticides (abamectin and beta-cypermethrin) through the silencing of *SfGSTD1* in *S. frugiperda* in vivo (Figure 3), the overexpression of *SfGSTD1* in *Sf9* cells in vitro (Figure 4), and the expression of *SfGSTD1* in transgenic fruit flies in vivo (Figure 5), respectively, although there are also results indicating that *SfGSTD1* (GeneBank: XP_035440580.2) has a strong binding ability to chlorantraniliprole, thereby regulating the tolerance of *S. frugiperda* to chlorantraniliprole [40]. However, through evolutionary tree analysis, we found that this gene is closely clustered with *SeGSTD3* (Figure S3), not the *SfGSTD1* (GeneBank: XP_050562830.1) in this study. In summary, we only found that *SfGSTD1* is responsible for the tolerance of abamectin and beta-cypermethrin. As the homologous gene of *GSTD1*, whether *SfGSTD1* could mediate the tolerance of *S. frugiperda* to other insecticides (such as chlorpyrifos, malathion, cyhalothrin, lambda-cyhalothrin and fenvalerate) needs further verification.

In insects, GST can not only directly metabolize insecticides, but also enhance their antioxidant stress activity or conjugation with insecticides, thereby mediating insect resistance to insecticides. For example, recombinant SeGSTo could protect super-coiled plasmid DNA from damage in the metal catalyzed oxidation system, indicating its involvement in the oxidative stress process of *S. exigua* [41]. GST genes detoxify organophosphorus insecticides by forming O-dearylation or O-dealkylation [42]. BdGSTD1 had the capacity to directly metabolize malathion, and could also catalyze the malathion reaction with the conjugation of reduced glutathione [32]. Although beta-cypermethrin was not directly metabolized by SlGSTD1, SlGSTE9, SlGSTE12 or SlGSTO2, these GST genes might play an important role in the tolerance *S. litura* to beta-cypermethrin through antioxidant and sequestration capabilities [37]. Similar to SlGSTD1, SlGSTE9, SlGSTE12 or SlGSTO2, CpGSTD1 also had glutathione peroxidase activity against substrate cumene hydroperoxide [19,37]. On the other hand, PaGSTD1 did not exhibit this activity, indicating that PaGSTD1 mainly promotes insecticide detoxification through metabolism rather than antioxidant capacity [38]. Furthermore, it was found that lambda-cyhalothrin could be quickly metabolized, but no new metabolites were discovered, suggesting that *CpGSTD3*, *CpGSTE3* and *CpGSTS2* are associated with the resistance of *C. pomonella* to lambda-cyhalothrin via sequestration [43]. Through inhibition experiments, we also found that the activity of SfGSTD1 could be significantly inhibited by abamectin and beta-cypermethrin (Figure 6), indicating that *SfGSTD1* might regulate the tolerance of *S. frugiperda* to these two insecticides through sequestration. Based on these previous research results, it can be inferred that even homologous genes (such as *GSTD1*) have different detoxification metabolic pathways in different insects. Therefore, whether *SfGSTD1* could exhibit antioxidant activity or directly metabolize insecticides such as abamectin and beta-cypermethrin remains to be verified through subsequent experiments.

## 5. Conclusions

In conclusion, we have cloned and characterized *SfGSTD1*, which is associated with the tolerance of *S. frugiperda* to abamectin and beta-cypermethrin. Temporal and spatial expression patterns showed that *SfGSTD1* was predominantly expressed in the Malpighian tubules, which were key tissues for insects to detoxify and metabolize insecticides. Functional analyses showed that the silencing of *SfGSTD1* reduced the tolerance of *S. frugiperda* to abamectin and beta-cypermethrin, the overexpression of *SfGSTD1* increased the viability of *Sf9* cells to abamectin and beta-cypermethrin and the overexpression of *SfGSTD1* in *D. melanogaster* decreased abamectin and beta-cypermethrin susceptibility. Moreover, the enzyme activity of SfGSTD1 was significantly inhibited by abamectin and beta-cypermethrin. All these results support the finding that *SfGSTD1* plays an essential role in the abamectin and beta-cypermethrin tolerance of *S. frugiperda*.

## Data Availability

The original contributions presented in this study are included in the article. Further inquiries can be directed to the corresponding author.

## Supplementary Materials

The following supporting information can be downloaded at https://www.mdpi.com/article/10.3390/insects16030299/s1, Figure S1: Amino acid sequence alignment of SfGSTD1; Figure S2: Expression and purification of recombinant *SfGSTD1*; Figure S3: Phylogenetic analysis of *SfGSTD1* and *SfGSTD3* gene; Table S1: Primers used in this study.
