# Supplementary material for: GSTD1 Mediates the Tolerance to Abamectin and Beta-Cypermethrin in the Fall Armyworm Spodoptera frugiperda
Source: Insects. 2025 Mar 12;16(3):299. doi: 10.3390/insects16030299 (PMC11943115; doi:10.3390/insects16030299)
Supplement: Supplementary file 1 [file insects-16-00299-s001.zip › insects-3501633-supplementary.pdf]

Supplementary Material for Review

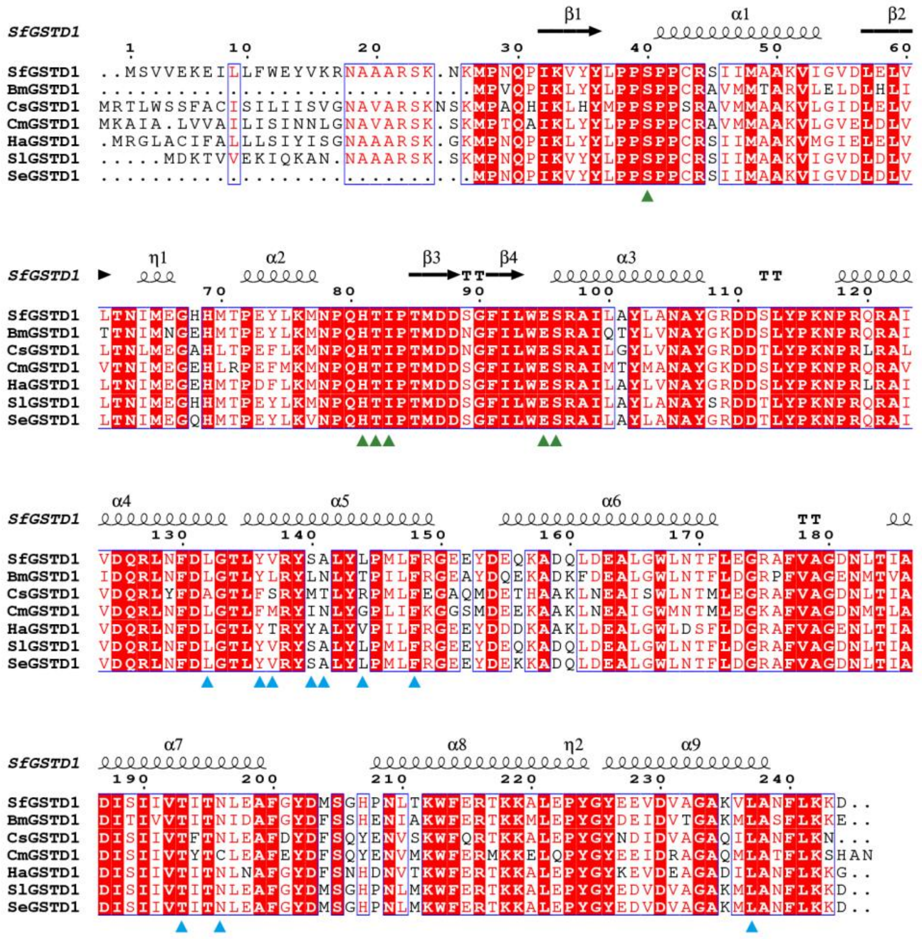

Figure S1. Amino acid sequence alignment of SfGSTD1 and orthologous proteins from *B. mori* (Bm); *C. medinalis* (Cm); *C. suppressalis* (Cs); *H. armigera* (Ha), *S. exigua* (Se); and *S. litura* (Sl). Conserved residues in orthologous sequences are displayed as white characters against red background. Six GSH-binding sites (G-site) and ten hydrophobic substrate-binding sites (H-site) were marked with green and blue triangles, respectively. The α-helix and β-sheet were marked with wavy lines.

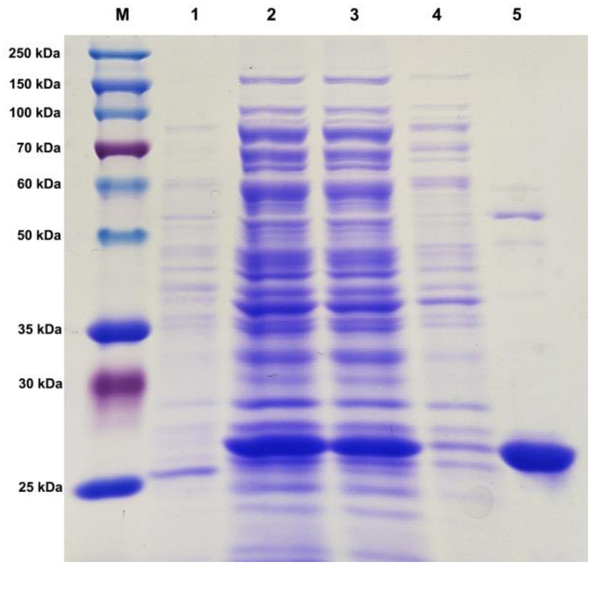

Figure S2. Expression and purification analysis of recombinant *SfGSTD1*. Lane M, protein molecular weight marker; lane 1, supernatant of empty vector control induced with 0.75 mM IPTG; lane 2, supernatant of the *SfGSTD1* fragment carrier induced with 0.75 mM IPTGa; lane 3, flow-through after column combination; lane 4, wash buffer fraction, and lane 5, purified recombinant SfGSTD1 protein.

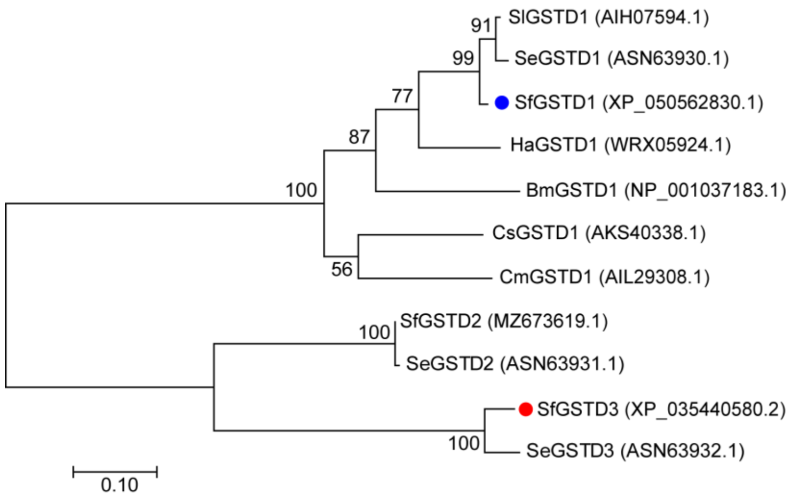

Figure S3. Phylogenetic analysis of *SfGSTD1* and *SfGSTD3* gene. The phylogenetic tree is constructed using maximum likelihood method. Accession numbers of the used sequences are listed after the corresponding genes. Bm, *B. mori*; Cm, *C. medinalis*; Cs, *C. suppressalis*; Ha, *H. armigera*; Se, *S. exigua*; Sf, *S. frugiperda*; Sl, *S. litura*.

Table S1. Primers used in this study.

Primers	Primer sequence (5′ - 3′)	Amplification efficiency (%)
For cDNA cloning		
SfGSTD1-F	ATGAGTGTTGTTGAAAAGGAGATA	
SfGSTD1-R	CTAATCTTTCTTCAAGAAGTTTGC	
For qPCR		
q-SfGSTD1-F	GATGCCAAACCAGCCAATCA	103.2
q-SfGSTD1-R	CGCGGCTCTCCCATAAAATG	
q-SfRPL32-F	TACAATCGTCAAAAAGAGGACGA	107.6
q-SfRPL32-R	AAACCATTGGGTAGCATGTGA	
q-SfGAPDH-F	CCGTTGACATGCAAGATGGC	95.0
q-SfGAPDH-R	AGACGCCTTCTCTGTGGTTG	
q-DmRp49-F	GCTAAGCTGTCGCACAAATG	95.6
q-DmRp49-R	GTTCGATCCGTAACCGATGT	
q-DmActin5C-F	CACACCAAATCTTACAAAATGTGTGA	99.1
q-DmActin5C-R	AATCCGGCCTTGCACATG	
For over-expression in Sf9		
bp-SfGSTD1-F	TCTATGCCTCTTAAAATCTAGCCAC-CATGAGTGTTGTTGAAAAGGA	
bp-SfGSTD1-R	ATAAACAAGTTAACGTCGAC-CTAATCTTTCTTCAAGAAGTT	
bp-EGFP-F	TCTATGCCTCTTAAAATCTAGCCACC	
bp-EGFP-R	ATAAACAAGTTAACGTCGAC	
For transgenic Drosophila		
26214-XhoI-SfGSTD1-F	TTCAGGCGGCCGCGGCTCGAG-CAAAATGAGTGTTGTTGAAAAGGAG	

26214-XbaI-SfGSTD1-R	CCTTCACAAAGATCCTCTA-GACTAATCTTTCTTCAAGAAGTT
For dsRNA synthesis	
PET2P-Not1-SfGSTD1-F	CTCCCGGCCGCCATGGCGGCCGCTGGGG-TAGACTTGGAACTCG
PET2P-Kpn1-SfGSTD1-R	CCCAAGGGGTTATGCTAGGGTACCAAAA-GCCTCAAGGTTCGTGA
PET2P-Not1-EGFP-F	CTCCCGGCCGCCATGGCGGCCGCGAT-TAAGTTCAGCGTGTCCG
PET2P-Kpn1-EGFP-R	CCCAAGGGGTTATGCTAGGGTACCAC-TAGTGATTCACCTTGATG
For recombination protein expression	
yh-FlGSTd7-F	TGCATCATCATCATCATCATATGAGTGTT-GTTGAAAAGGAGATA
yh-FlGSTd7-R	TGCTTTTAAGCAGAGATTAC-CTAATCTTTCTTCAAGAAGTTTGC
